# Effect of Childhood Victimization on Occupational Prestige and Income Trajectories

**DOI:** 10.1371/journal.pone.0115519

**Published:** 2015-02-27

**Authors:** Cristina A. Fernandez, Sharon L. Christ, William G. LeBlanc, Kristopher L. Arheart, Noella A. Dietz, Kathyrn E. McCollister, Lora E. Fleming, Carles Muntaner, Peter Muennig, David J. Lee

**Affiliations:** 1 Department of Public Health Sciences, University of Miami Miller School of Medicine, Miami, Florida, United States of America; 2 Department of Human Development and Family Studies; Department of Statistics, Purdue University, West Lafayette, Indiana, United States of America; 3 Division of Biostatistics, Department of Epidemiology and Public Health, University of Miami Miller School of Medicine, Miami, Florida, United States of America; 4 European Centre for Environment and Human Health, University of Exeter Medical School; Knowledge Spa, Royal Cornwall Hospital, Cornwall, United Kingdom; 5 Bloomberg Faculty of Nursing; Dalla Lana School of Public Health; Department of Psychiatry, University of Toronto, Toronto, Ontario, Canada; 6 Department of Health Care Management, Korea University, Seoul, South Korea; 7 Department of Health Policy and Management, Mailman School of Public Health, Columbia University, New York, New York, United States of America; Universität Bochum, GERMANY

## Abstract

**Background:**

Violence toward children (childhood victimization) is a major public health problem, with long-term consequences on economic well-being. The purpose of this study was to determine whether childhood victimization affects occupational prestige and income in young adulthood. We hypothesized that young adults who experienced more childhood victimizations would have less prestigious jobs and lower incomes relative to those with no victimization history. We also explored the pathways in which childhood victimization mediates the relationships between background variables, such as parent’s educational impact on the socioeconomic transition into adulthood.

**Methods:**

A nationally representative sample of 8,901 young adults aged 18–28 surveyed between 1999–2009 from the *National Longitudinal Survey of Youth 1997* (NLSY) were analyzed. Covariate-adjusted multivariate linear regression and path models were used to estimate the effects of victimization and covariates on income and prestige levels and on income and prestige trajectories. After each participant turned 18, their annual 2002 Census job code was assigned a yearly prestige score based on the 1989 General Social Survey, and their annual income was calculated via self-reports. Occupational prestige and annual income are time-varying variables measured from 1999–2009. Victimization effects were tested for moderation by sex, race, and ethnicity in the multivariate models.

**Results:**

Approximately half of our sample reported at least one instance of childhood victimization before the age of 18. Major findings include 1) childhood victimization resulted in slower income and prestige growth over time, and 2) mediation analyses suggested that this slower prestige and earnings arose because victims did not get the same amount of education as non-victims.

**Conclusions:**

Results indicated that the consequences of victimization negatively affected economic success throughout young adulthood, primarily by slowing the growth in prosperity due to lower education levels.

## Introduction

Violence toward children—whether arising from adults or bullying by other children—negatively affects victims over their entire life course.[1] Examples of long-term consequences of this violence, which is commonly referred to as “childhood victimization,” include poor mental/physical health,[2,3] substance use, productivity losses, and criminal behavior.[4,5] Relative to adults without a history of childhood victimization, adults with this history may also be more likely to have educational underachievement,[6] higher unemployment, and be employed in unskilled/semi-skilled jobs rather than skilled/professional jobs.[7]^,^[6] Additionally, the total lifetime burden of nonfatal childhood victimization in the US has been estimated to be approximately $124 billion in one year.[5] Considering violence towards children has proven to be a major public health problem and its negative effects tend to cumulate over time, there remains a lack of research investigating its longitudinal effects on future “occupational prestige.”[8]

Occupational prestige is a sociological construct that measures the shared belief about the “social standing” of a profession.[9]^,^[10,11] For example, doctors score high on the occupational prestige scale, whereas truck drivers score low. However, a high-prestige job does not necessarily imply that it is highly paid. For example, teachers and firefighters have relatively high prestige scores despite a relatively low income.[12,13] Even though it is less commonly used than income or education in research settings,[11] occupational prestige is a reliable measure of society’s perceptions about success/social standing across generations and a powerful indicator of socioeconomic status.[10,14] Similarly, income is considered a marker for an individual’s relative societal standing, and is associated with current living standards and future social opportunities.[15,16]

Although some studies have reported an association between childhood victimization and lower earnings in adulthood,[17,18] very little is known about the negative effects of childhood victimization on occupational prestige. To the authors’ knowledge, only one previous study has examined childhood victimization and occupational prestige during 1976–1986.[8] This study used Duncan’s Scale of Occupational Status, which was based on age-standardized income and education levels of males from the 1950 Census. This scale is simply a weighted average of education and income and is not currently considered a measure of occupational prestige.[19] Additionally, because this scale ignores women, does not take into account racial variations, and does not include modern professions, it is now considered outdated.[9,20]^,^[21] As the population of racial/ethnic minorities in the US continues to rise, it is important to include these diverse subgroups in prospective longitudinal studies, as their socioeconomic impacts of victimization are unclear.

The objective of this study was to determine if victimization during childhood affects two different measures of SES in young adulthood, occupational prestige and income. Because both childhood victimization and socioeconomic status are life course phenomena, we also explored the pathways in which childhood victimization mediates the relationships between background variables, including parent’s educational impact of the socioeconomic transition into adulthood.[6,8,17,22–29] We hypothesized that young adults who experienced more victimization during their childhood would have less prestigious jobs and lower incomes relative to their counterparts with no victimization history. We also hypothesized that there will be differences in their trajectories over time, in terms of occupational prestige and income as a function of childhood victimization history.

## Methods

### National Longitudinal Survey of Youth 1997 (NLSY97)

Data were analyzed from the NLSY97, a nationally representative prospective survey of 8,984 youth that examines topics of employment/educational history, family background, and numerous other areas, designed to document the transition from school to work and into adulthood.[30–32] Youth aged 12–16 at baseline and their parents were first surveyed in 1997, and have been interviewed annually through 2011, at which time respondents were aged 25–29.[33] The retention rates for 1998–2011 were 93.3%, 91.4%, 89.9%, 87.7%, 87.9%, 86.3%, 83.5%, 81.7%, 84.1%, 82.6%, 83.3%, 84.1%, 83.2%, and 82.6%, respectively.[34] More information regarding the NLSY97 is publicly available.[30]

### Analytic Sample

We evaluated 8,901 unique respondents between the survey years 1999–2009. These individuals were 14–18 years old at our study baseline (i.e., 1999), and were 24–28 years old at the end of our study follow-up (i.e., 2009). Each participant had observations at one or more time points. Participants were included each year that they were both 18+ years and participated in the survey. Consequently, we evaluated a total of 80,018 person-by-time observations. Participants missing values for some model variables are included in analysis using direct maximum likelihood for item missing.[35] The number of individuals per year ranged from 847 to 6,697.

### Childhood Victimization

We calculated the victimization variables following the approach used by Boynton-Jarrett et al (2008).[36] Childhood victimization measures victimization events prior to age 18 in five areas: victim of violent crime (e.g., physical or sexual assault, robbery, or arson), bullying (i.e., victim of repeated bullying), gun violence (e.g., ever been shot at, or seen someone get shot or shot at with a gun), perceived school safety (i.e., “Do you feel safe at school?”), and threatened violence at school (e.g., if someone had threatened to hurt participant). The school-related questions, perceived safety and threatened violence, were asked in 1997 when respondents were in school, with each counting as one victimization type. The bullying and gun violence events were asked retrospectively after respondents turned 18; and included a count for an early childhood occurrence (prior to age 12) and a count for an adolescent occurrence (age 12 to 18). Victim of violent crime was reported retrospectively when respondents were 18 years old or older, and referred to events occurring during adolescence. An affirmative response was counted only once for crime victimization. Due to the low number of participants reporting victimizations, we summed the number of victimization experiences to create a “victimization score,” ranging from 0–7.[36]

### Occupational Prestige

After each participant turned 18, we used his/her yearly 2002 Census job code to assign an occupational prestige score based on the 1989 General Social Survey (GSS).[10,37] We converted the Census job codes into the prestige scores using a publicly available occupational crosswalk.[38] To develop the occupational prestige scores in the GSS, respondents evaluated 740 randomly ordered occupation titles, and rated them from 1–9 based on perceived social standing.[9,10] Prestige scores were calculated by taking a weighted mean score of the ratings for an occupation, where the weights rescale the means to range from 0 (lowest score) to 100 (highest score).[9,10] The GSS samples are representative of the US population; therefore, the scores are an unbiased estimate of the prestige evaluations.[9,10] For participants with multiple jobs/year, we used the maximum prestige score for that year. Prestige scores are a time-varying variable measured from 1999–2009.

### Annual Income

After each participant turned 18, we used their self-reported yearly income. This measure did not include parental/household income. For participants reporting different levels of earning within one year, we used their maximum income. Annual income is a time varying variable measured from 1999–2009.

### Confounders

Selected confounders were based on previous literature and theory[39,40] [4–7] [17,18], [8] ^and references therein^:sex, race, ethnicity, and age, all measured in 1999. The number of years participants had two biological parents in the house was summed for each year that the participant was less than 18 years old and observed in that year. All models also controlled for the highest educational grade completed of both the participant and their parents, and the participant’s school enrollment status. Participant highest grade completed and enrollment status were time-varying covariates.

### Statistical Analyses

Multivariate linear regression and path models were used to obtain effect estimates. Multiple observations per person were evaluated, with time-varying prestige and income outcomes observed after respondents had turned 18. Multivariate regression models were estimated with the occupational prestige and income outcomes being allowed to correlate (i.e., seemingly unrelated regressions). The first model evaluated the role that victimization played on levels of prestige and income during young adulthood (i.e., pooled across time).

The effects of victimization on these outcomes were tested for moderation by participants’ sex, race, and ethnicity. This model is outlined in the following equation:

$$\begin{array}{l} y_{\mathrm{it}}=\alpha \;+\;\beta_{1}{\text{male}}_{i}\;+\;\beta_{2}{\text{age}}_{i}\;+\;\beta_{3}{\text{black}}_{i}\;+\;\beta_{4}{\text{Hispanic}}_{i}\;+\;\beta_{5}{\text{bio parents}}_{i}\;+\;\beta_{6}{\text{parent education}}_{i}\\ +\;\beta_{7}{\text{respondent education}}_{\mathrm{it}}\;+\;\beta_{8}{\text{enrollment}}_{\mathrm{it}}\;+\;\beta_{9}{\text{victimization}}_{i}\;+\;\beta_{10}{\text{male}}_{i}{\text{Xvictimization}}_{i}\\ +\;\beta_{11}{\text{black}}_{i}{\text{Xvictimization}}_{i}\;+\;\beta_{12}{\text{Hispanic}}_{i}\text{X}\;{\text{victimization}}_{i}\;+\;\varepsilon_{\mathrm{it}}. \end{array}$$

for each individual, i:

There are two *y*_*it*_, income and prestige

Male_i_ = male sex

age_i_ = age at baseline

black_i_ = black race

Hispanic_i_ = Hispanic ethnicity

parents_i_ = cumulative years of two biological parents in the house before age 18

parent education_i_ = maximum number of years of education of father or mother

respondent education_it_ = respondent’s highest grade completed

enrollment_it_ = proportion of participants in any post-secondary education

victimization_i_ = overall victimization score ≤ 18 years

Interactions that were not statistically significant were removed from the final model.

The second model evaluated how victimization impacted both the baseline levels (1999) and the average annual change in prestige and income over time (i.e., the trajectories). In the trajectory analyses, income and prestige were evaluated in terms of time measured in years. The linear and quadratic effects of time were tested; and interactions of victimization with time were tested to determine whether victimization affected changes in prestige and income across the 11 survey years. We also tested if the effects of victimization on the baseline levels and change over time, moderated by the participants’ sex, race, and ethnicity due to differences related to the prevalence, impact, and response to victimization[41–43] as well as racial and ethnic differences in socioeconomic attainment. The trajectory model equation is as follows:

$$\begin{array}{l} y_{\mathrm{it}}=\alpha \;+\;\beta_{1}{\text{male}}_{i}\;+\;\beta_{2}{\text{age}}_{i}\;+\;\beta_{3}{\text{black}}_{i}\;+\;\beta_{4}{\text{Hispanic}}_{i}\;+\;\beta_{5}{\text{bio parents}}_{i}\;+\;\beta_{6}{\text{parent education}}_{i}\\ +\;\beta_{7}{\text{respondent education}}_{\mathrm{it}}\;+\;\beta_{8}{\text{enrollment}}_{\mathrm{it}}\;+\;\beta_{9}{\text{victimization}}_{i}\;+\;\beta_{10}{\text{male}}_{i}{\text{Xvictimization}}_{i} \end{array}$$

$$+\;\beta_{11}{\text{black}}_{i}{\text{Xvictimization}}_{i}\;+\;\beta_{12}{\text{Hispanic}}_{i}{\text{Xvictimization}}_{i}\;+\;\beta_{13}{\text{year}}_{\mathrm{it}}\;+\;\beta_{14}{\text{year}}_{\mathrm{it}}{\text{Xyear}}_{\mathrm{it}}$$

$$+\;\beta_{15}{\text{victimization}}_{i}{\text{Xyear}}_{\mathrm{it}}\;+\;\beta_{16}{\text{male}}_{i}{\text{Xyear}}_{\mathrm{it}}\;+\;\beta_{17}{\text{black}}_{i}{\text{Xyear}}_{\mathrm{it}}\;+\;\beta_{18}{\text{Hispanic}}_{i}{\text{Xyear}}_{\mathrm{it}}\;+$$

$$\begin{array}{l} \beta_{19}{\text{victimization}}_{i}{\mathrm{Xyear}}_{\mathrm{it}}{\text{Xyear}}_{\mathrm{it}}\;+\;\beta_{20}{\text{male}}_{\text{i}}{\text{Xvictimization}}_{i}{\text{Xyear}}_{\mathrm{it}}\;+\\ \beta_{21}{\text{black}}_{i}{\text{Xvictimization}}_{i}{\text{Xyear}}_{\mathrm{it}}\;+\;\beta_{22}{\text{Hispanic}}_{i}{\text{Xvictimization}}_{i}{\text{Xyear}}_{\mathrm{it}}\;+\;\varepsilon_{\mathrm{it}}. \end{array}$$

where one *y*_*it*_ is income and one is prestige. The other labels follow those of the first model. The quadratic effect of year and interactions involving participants’ sex, race, and ethnicity that were not statistically significant were removed from the final model. Although not included in the equation, interactions between sex, race, and ethnicity with the quadratic effect of year were also tested for the prestige outcome, which exhibited a quadratic effect for year.

**Fig 1:**
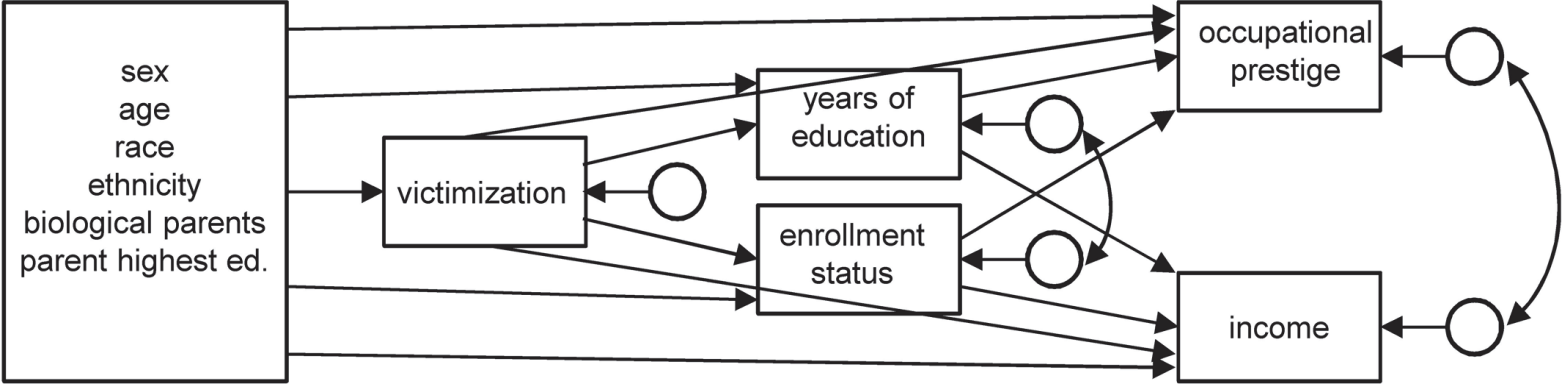
Path model assessing pathways by which parent’s education and respondent’s childhood victimization impact occupational prestige and income.

In a final model, rather than control for respondent’s education and school enrollment status when looking at the effects of victimization, we evaluated a path model in which respondent demographics and parent education affected childhood victimization, which in-turn impacts respondents’ education and school enrollment status, which in-turn impact on their occupational prestige and income. Fig. 1 portrays this path model. We evaluated these relationships within time looking at effects on prestige and income levels.

In all models, independent variables are mean-centered except for year, which is centered at baseline (1999). All statistical analyses were performed using SAS v9.2.[44] and MPlus v.7.0.[45] All estimates were weighted to correct for the unequal selection probabilities in the NLSY97; standard errors were adjusted for the weights and the nesting of time within persons using between cluster variance estimation. The University of Miami IRB reviewed and approved this study.

## Results

### Demographic Information

Demographic information is presented in Table 1. There were slightly more males than females, and the majority of the sample was non-Hispanic whites. Half of the participants had a victimization experiences (50.5%), 25.8% had one occurrence, 14.3% had two, 6.6% had three, 2.8% had four, and the remaining <1% of respondents had 5–7 experiences (results not shown). There were statistically significant differences (p<0.001) in the mean number of victimizations between males (1.01) and females (0.80), and between blacks (1.21) and whites (0.85; see Table 1).

**Table 1:** Demographic Information of Sample: The National Longitudinal Survey of Youth, 1999–2009 (N = 8,901).

**Variable**	*Mean (Standard Error)*	*95% Confidence Interval*
Age in 1999 (centered)	14.00 (0.02)	13.97–14.04
^a^Male	0.51 (0.01)	0.50–0.52
^a^Black	0.16 (0.00)	0.15–0.16
^a^Hispanic	0.13 (0.00)	0.12–0.14
Overall Victimization Score ≤ 18 years	0.91 (0.01)	0.88–0.94
Victimization by race and gender		
Male (n = 3,906)	1.01 (0.02)	0.97–1.05
Female (n = 3,834)	0.80 (0.02)	0.76–0.84
White (n = 4,063)	0.85 (0.02)	0.81–0.88
Black (n = 2,041)	1.21 (0.03)	1.15–1.27
Hispanic (n = 1,636)	0.88 (0.03)	0.83–0.94
^b^Biological parents	2.95 (0.03)	2.88–3.02
^c^Parent Highest Education	13.65 (0.04)	13.58–13.72
Respondent Highest Grade	12.35 (0.03)	12.29–12.41
^d^Student Enrollment Status	0.35 (0.00)	0.34–0.36
Respondent Occupational Prestige Score	40.78 (0.11)	40.57–40.99
Respondent Income	$22,463 ($185)	$22,100–$22,827

Note: n’s were calculated at baseline (1999); Victimization score and biological parents weremeasured before the participant turned 18; income, and prestige were measured over the entire survey period and are time-varying

^a^Proportions of each group in sample

^b^Cumulative years of two biological parents in the house before age 18

^c^Maximum number of years of education of father or mother

^d^Proportion of participants in any post-secondary education over the 11 year (i.e., persons could be enrolled in more than one year)

In general, as the study population aged, their occupational prestige and income increased (see Table 2). Prestige increased by an average of 0.86 points per year (p<0.001) and income increased by an average of $2,873 per year across the 11 years (p<0.001; results not shown).

**Table 2:** Yearly Occupational Prestige Scores and Income of Sample: The National Longitudinal Survey of Youth, 1999–2009.

**Variable**	*Mean (Standard Error)*	*95% Confidence Interval*
**Occupational Prestige**		
1999 (n = 1,291)	36.5 (0.3)	35.9–37.1
2000 (n = 2,742)	38.5 (0.2)	38.0–38.9
2001 (n = 4,033)	38.6 (0.2)	38.2–38.9
2002 (n = 5,388)	38.8 (0.2)	38.5–39.2
2003 (n = 6,627)	39.1 (0.2)	38.8–39.4
2004 (n = 6,491)	40.6 (0.2)	40.3–40.9
2005 (n = 6,409)	41.8 (0.2)	41.5–42.2
2006 (n = 6,697)	43.1 (0.2)	42.8–43.4
2007 (n = 6,578)	44.0 (0.2)	43.7–44.4
2008 (n = 6,635)	44.5 (0.2)	44.2–44.9
2009 (n = 6,427)	44.6 (0.2)	44.2–44.9
**Income**		
1999 (n = 847)	$9,027 ($293)	$8,451–$9,602
2000 (n = 1,889)	$10,212 ($217)	$9,787–$10,637
2001 (n = 2,612)	$11,571 ($206)	$11,166–$11,975
2002 (n = 3,580)	$12,520 ($187)	$12,154–$12,886
2003 (n = 4,268)	$14,747 ($219)	$14,318–$15,176
2004 (n = 4,349)	$17,851 ($226)	$17,408–$18,294
2005 (n = 4,684)	$21,410 ($252)	$20,916–$21,904
2006 (n = 4,869)	$25,662 ($310)	$25,054–$26,270
2007 (n = 5,143)	$28,903 ($310)	$28,295–$29,511
2008 (n = 5,378)	$31,806 ($344)	$31,133–$32,480
2009 (n = 5,037)	$33,564 ($385)	$32,809–$34,320

However, the change in occupational prestige was non-linear, with greater increases occurring in earlier years and smaller increases occurring in later years.

### Pooled Time Multivariate Model

The association of victimization experiences with prestige scores was moderated by sex, where there is no association for females within time after controlling for the aforementioned covariates (p = 0.305; Table 3). There was a significant difference between males and females in the way that victimization impacted prestige (Sex X Victimization = 0.44, p < 0.01). Males had a *positive* association between the number of victimizations experienced in childhood and their prestige score (β = 0.53 [= 0.09 + 0.44]; p < 0.01).

**Table 3:** Multivariate Linear Regression of the Effect of Victimization on Occupational Prestige and Income: The National Longitudinal Survey of Youth, 1999–2009 (N = 80,018 time points nested in 8,901 persons).

	**Pooled Prestige Levels**		**Pooled Income Levels**	
**Variable**	*β (Standard Error)*	*P-value*	*β (Standard Error)*	*P-value*
Intercept	**40.09 (0.09)**	<.001	**$18,780 ($1,350)**	<.001
Male Sex	**-0.63 (0.20)**	.001	**$6,667 ($344)**	<.001
Age	**0.38 (0.07)**	<.001	**$912 ($123)**	<.001
Race/Ethnicity				
White	Ref	-		
Black	**-1.72 (0.24)**	<.001	**-$3,534 ($372)**	<.001
Hispanic	-0.33 (0.26)	.203	-$428 ($424)	.313
^a^Biological parents	0.01 (0.04)	.827	-$97 ($63)	.123
^b^Parent Highest Education	**0.22 (0.04)**	<.001	**-$184 ($68)**	<.01
Respondent Highest Education	**2.24 (0.05)**	<.001	**$2,783 ($87)**	<.001
^c^Student Enrollment Status	**-3.53 (0.17)**	<.001	**-$15,818 ($331)**	<.001
Victimization Score ≤ 18 years	0.09 (0.09)	.305	**-$301 ($154)**	.05
Sex X Victimization	**0.44 (0.16)**	<.01	-	-

^a^Cumulative years of two biological parents in the house before age 18

^b^Maximum number of years of education of father or mother

^c^Proportion of participants in any post-secondary educationNote: X indicates multiplication and is used to describe interaction effects. Variables interacted withthemselves (e.g., sex X victimization) represent non-linear effects of those predictors on outcomes.

For both genders, victimization had a negative association with income levels, where an additional victimization experience in childhood was associated with $301 lower income per year (p = 0.05). The effect of victimization on income did not differ across sex, race, or ethnicity, that is, was not moderated.

Several covariates were related to prestige and income. Notably, being a college student, relative to not being a college student, was related to a 3.53 lower prestige score (p <0.001) and $15,818 lower income (p < 0.001). A year of additional education of the participant was associated with a 2.24 higher prestige score (p <0.001) and $2,783 higher income (p <0.001). The correlation of the residual error for prestige and income was 0.20 (p < 0.001) in this model.

### Multivariate Trajectory Model

Main effects in this model represent effects within time at baseline (1999) for the average individual. Most results for these effects were similar to the pooled model. Prestige increased by 0.99 (p < 0.001) per year on average as the participants aged, but this slowed over time. Every year, the increase went down by 0.05 points (p <0.001; Table 4). Victimization experiences in childhood were positively related to prestige at baseline; an additional victimization experience was associated with an increase of 0.65 (p < 0.001) in prestige. However, victimization experiences were related to smaller increases over time, such that for each additional victimization experience, prestige scores gained 0.10 points less annually (p < 0.001). Therefore, after 6.5 years, the higher prestige score associated with victimization at baseline disappeared, on average.

**Table 4:** Multivariate Linear Regression of the Effect of Victimization on Changes in Prestige and Annual Income: The National Longitudinal Survey of Youth, 1999–2009 (N = 80,018 time points nested in 8,901 persons).

	**Prestige Trajectories**		**Income Trajectories**	
**Variable**	*β (Standard Error)*	*P-value*	*β (Standard Error)*	*P-value*
Intercept	**36.60 (0.24)**	<.001	**$4,859 ($256)**	<.001
Male Sex	**-0.67 (0.20)**	.001	**$6,191 ($340)**	<.001
Age	**0.67 (0.08)**	<.001	**$2,327 ($134)**	<.001
Race/Ethnicity				
White	Ref	-		
Black	**-1.78 (0.24)**	<.001	**-$3,858 ($371)**	<.001
Hispanic	-0.40 (0.25)	.119	**$1,569 ($493)**	.001
^a^Biological parents	0.03 (0.04)	.498	$60 ($62)	.336
^b^Parent Highest Education	**0.24 (0.04)**	<.001	-$10 ($68)	.888
Respondent Highest Education	**2.05 (0.05)**	<.001	**$1,561 ($90)**	<.001
^c^Student Enrollment Status	**-2.51 (0.18)**	<.001	**-$9,670 ($306)**	<.001
Victimization Score ≤ 18 years	**0.65 (0.13)**	<.001	**$1,003 ($175)**	<.001
Year	**.99 (0.09)**	<.001	**$2,349 ($46)**	<.001
Year X Year	**-.05 (0.01)**	<.001	-	-
Year X Victimization	**-.10 (0.02)**	<.001	**-$259 ($33)**	<.001
Sex X Victimization	**.43 (0.16)**	<.01	-	-
Hispanic X Victimization	-	-	**-$1,222 ($391)**	.002
Hispanic X Year	-	-	**-$403 ($85)**	<.001
Year X Victimization X Hispanic	-	-	**$199 ($72)**	.006

^a^Cumulative years of two biological parents in the house before age 18

^b^Maximum number of years of education of father or mother

^c^Proportion of participants in any post-secondary educationNote: X indicates multiplication and is used to describe interaction effects. Variables interacted with themselves (e.g., year X year) represent non-linear effects of those predictors on outcomes.

Income linearly increased by $2,349 (p <0.001) on average every year. Hispanics were earning more at baseline, but also had lower income growth (less $403 per year) over time (i.e., Hispanic X Victimization). Victimization was associated with $1,003 more in annual income at baseline (p <0.001). This association was not evident for Hispanics, who experienced no change in income at baseline due to victimization (β = -$219 [= 1,003–1,222]; p = 0.60). Additional childhood victimization experiences were associated with lower growth in income over time. Every year, an additional victimization experience was associated with $259 lower income growth (p <0.001) annually. Therefore, after four years, the higher income at baseline associated with victimization was eroded, and continued to stagnate relative to those with fewer victimization experiences. The loss of income over time, due specifically to victimization, was not experienced by Hispanics (-$60 [= -259 + 199]; p = 0.42). The correlation of the residual error for prestige and income was 0.18 (p < 0.001) in this model.

### Multivariate Path Model

The direct effects of variables in the model on occupational prestige and income were virtually identical to those from Model 1 (Table 2). Relevant indirect effects are presented in Table 5. The effects of parent highest education on occupational prestige and income through victimization pathways were very small, even when statistically significant. The largest indirect effects were the impact of victimization through highest educational level completed; an additional childhood victimization experience was related to a 0.84 lower prestige score (p < 0.001) and $1,036 lower income (p < 0.001). Victimization had a positive impact on prestige and income through student enrollment status due to a negative association between victimization and enrollment, and a negative association between enrollment and both occupational prestige and income. The total effects of parental highest education and victimization (direct plus indirect effects) were statistically significant, but not very large effect sizes. The indirect effects of victimization through education were larger. For example, an additional childhood victimization experience was associated with 0.62 lower prestige score (p < 0.001) and the loss of income due to victimization through all pathways was -$891 (p < 0.001).

**Table 5:** Multivariate Path Model of the Indirect and Total Effects of Parent Highest Education and Respondent Victimization on Prestige and Annual Income: The National Longitudinal Survey of Youth, 1999–2009 (N = 80,018 time points nested in 8,901 persons).

	**Prestige**		**Income**	
**Indirect Effects**	*β (Standard Error)*	*P-value*	*β (Standard Error)*	*P-value*
Parent Highest Education -> Victimization	-0.003(0.003)	0.226	$8 ($5)	0.061
Parent Highest Education -> Victimization -> Years of Education	0.023 (0.005)	< 0.001	$29 ($6)	< 0.001
Parent Highest Education -> Victimization -> Student Enrollment Status	-0.003 (0.001)	< 0.001	-$12 ($3)	< 0.001
Victimization -> Years of Education	-0.835 (0.054)	< 0.001	-$1,036 ($71)	< 0.001
Victimization -> Student Enrollment Status	0.100 (0.010)	< 0.001	$448 ($40)	< 0.001
**Total Effects**				
Parent Highest Education	0.793 (0.040)	< 0.001	$175 ($67)	0.009
Victimization	-0.624 (0.095)	< 0.001	-$891 ($153)	< 0.001

## Discussion

Major findings of the current study indicate that: 1) childhood victimization resulted in slower income and prestige growth over time, and 2) mediation analyses suggested that this slower prestige and earnings arose because victims did not get the same amount of education as non-victims. It is important to note that these results were demonstrated despite controlling for other socioeconomic measures, including educational attainment and income and prestige, respectively. This likely yielded small effect sizes in our models. Because victimized children are at an increased risk of educational underachievement and behavioral/psychological problems, it is not surprising that the consequences of victimization negatively affected economic prosperity throughout young adulthood in this study population.[7,18,26,46,47]

Surprisingly, in the pooled occupational prestige models, victimization was not associated with prestige among females. This is inconsistent with other studies that have found women to have more difficulty coping with victimization compared to men,[18,48–52] which may be due to increased risk of re-victimization during adulthood,[48] tendency to internalize issues, and higher risk of developing psychopathological reactions to being victimized.[50,51] However, the impact of victimization on occupational prestige for females is significantly more negative compared to males for whom victimization was positively associated with prestige. These results among males may be reflective of the “inoculation hypothesis,” which posits that exposure to stress increases resistance to subsequent stress, thus ultimately protecting individuals.[53] Therefore, males in our sample with several victimizations may have been “inoculated” from other types of exposures to victimization. In the prestige trajectory models however, victimization negatively affected the growth potential of occupational prestige over time in both sexes. This may be because childhood victimization has been linked to several behaviors that limit occupational growth potential, such as: lower productivity, more frequent tardiness/absenteeism, job turnover/termination, and fewer hours worked.[39,40]

In the pooled income models, all groups in our sample had incomes that were $301/year lower for each victimization occurrence. This is consistent with previous literature that has found abused children to have lower earnings.[18] Additionally, victimization slowed the growth of annual income/year in both sexes in the trajectory models. Of note, while the amount of money lost in the income analyses may not be considered large for an older individual more advanced in his/her career, it is important to consider the relative amount of income lost for this age group and the even larger amount of accumulated income lost over the lifetime, especially if long-term gains and compound increases are taken into account. For example, after the first 10 years of labor force participation, non-Hispanic young adults who experienced three victimizations made almost $8,000 less/year compared to those with no victimization. These losses have additional consequences, such as on the building of equity and intergenerational transmission of wealth.

In the path analyses, higher parental education attainment reduced childhood victimization,[23,24] increased childhood victimization decreased the educational attainment of the participants,[6,25,26] and higher educational attainment of the participants increased both prestige and income.[27] Overall, these results are similar to other researchers who have found similar pathways; slower prestige and earnings arose because victims did not get the same amount of education as non-victims.[8,17,29]

### Strengths and Limitations

Our study has some limitations that should be noted when interpreting results. First, while our study attempted to control for key determinants of occupational prestige and income, several potentially important variables (such as mental health) were not included in the analyses due to high rates of missing data. Second, although the NLSY97 database is prospective, it relies on young adults accurately recalling and reporting all information related to victimization experiences, presenting the possibility of recall bias. It is well known that victims of violence do not always openly discuss their experiences due to a variety of reasons, such as stigma or fear of retaliation; therefore, some participants may have not answered these questions honestly. Third, due to some questions with few responses, we had to combine diverse victimization experiences into a single victimization score.[36] This may not accurately reflect the negative and potentially different impacts of specific types of victimization. Fourth, there is potential for selection bias in our sample. Although we included most individuals in at least one wave, there was some attrition over time. Additionally, the most severely victimized who may have consequently died or were not employed were not included in our sample. Therefore, our results are likely not generalizable to the most severely impacted by childhood victimization. Fifth, many of our participants were still in school or just starting in the paid labor force when their income and prestige was calculated, which may have underestimated the actual long-term effects of childhood victimization. Because of this, it is possible that some individuals in our sample were still financially dependent on their parents. Finally, these jobs may not represent the prestige or income of their jobs when they are fully integrated into the labor force. Future longitudinal studies should examine the socioeconomic outcomes of adults throughout their life course to obtain a more accurate picture of the effects of childhood victimization, including the mechanisms for which these consequences occur.[18]

Despite these limitations, our study used a prospective longitudinal nationally representative sample of youth to examine the unique socioeconomic effects of childhood victimization. To the authors’ knowledge, this is the first modern study to examine the longitudinal effects of victimization on occupational prestige. The large nationally representative sample and adequate number of follow-up years of the NLSY97 allowed for precise and externally valid estimates, and moderation testing of race/ethnic and gender sub-populations. Additionally, we were able to disentangle the pathways in which childhood victimization affected economic prosperity in adulthood, a necessary first step before interventions or policy changes can occur. With this information, it becomes possible to assess the magnitude of the investments needed to address this problem with effective policies. Future studies should examine other economic outcomes to guide policy recommendations, such as job turnover and part-time worker status in those victimized during childhood.

## Conclusions

The current study refines the understanding of how adults’ socioeconomic trajectories are negatively affected by childhood victimization. While there is a recent increased awareness of bullying, child abuse, and other forms of victimization in the US, more aggressive, and perhaps expensive, policies may prove to be superior to the current status quo. Understanding the effects of childhood victimization provides information regarding its long-term economic consequences.
